# Longitudinal multi-omics analysis identifies early blood-based predictors of anti-TNF therapy response in inflammatory bowel disease

**DOI:** 10.1186/s13073-022-01112-z

**Published:** 2022-09-24

**Authors:** Neha Mishra, Konrad Aden, Johanna I. Blase, Nathan Baran, Dora Bordoni, Florian Tran, Claudio Conrad, Diana Avalos, Charlot Jaeckel, Michael Scherer, Signe B. Sørensen, Silja H. Overgaard, Berenice Schulte, Susanna Nikolaus, Guillaume Rey, Gilles Gasparoni, Paul A. Lyons, Joachim L. Schultze, Jörn Walter, Vibeke Andersen, SYSCID Consortium, Aggelos Banos, George Bertsias, Marc Beyer, Dimitrios Boumpas, Axel Finckh, Andre Franke, Michel Georges, Wei Gu, Robert Häsler, Mohamad Jawhara, Amy Kenyon, Christina Kratsch, Roland Krause, Gordan Lauc, Massimo Mangino, Gioacchino Natoli, Marek Ostaszewski, Marija Pezer, Jeroen Raes, Souad Rahmouni, Marilou Ramos-Pamplona, Benedikt Reiz, Elisa Rosati, Despina Sanoudou, Venkata Satagopam, Reinhard Schneider, Jonas Schulte-Schrepping, Prodromos Sidiropoulos, Kenneth G. C. Smith, Timothy Spector, Doris Vandeputte, Sara Vieira-Silva, Aleksandar Vojta, Stefanie Warnat-Herresthal, Vlatka Zoldoš, Emmanouil T. Dermitzakis, Stefan Schreiber, Philip Rosenstiel

**Affiliations:** 1 Institute of Clinical Molecular Biology, Christian-Albrechts-University Kiel and University Medical Center Schleswig-Holstein, Kiel, Germany; 2 Department of Internal Medicine I, University Medical Center Schleswig-Holstein, Kiel, Germany; 3 Institute of Genetics and Genomics of Geneva (iGE3), University of Geneva, Geneva, Switzerland; 4 Department of Genetics, University of Saarland, Saarbrücken, Germany; 5 Present address: Department of Bioinformatics and Genomics, Centre for Genomic Regulation, The Barcelona Institute of Science and Technology, 08003 Barcelona, Spain; 6 The Molecular Diagnostics and Clinical Research Unit, University Hospital of Southern Denmark, Aabenraa, Denmark; 7 Institute of Molecular Medicine, University of Southern Denmark, Odense, Denmark; 8 Section for Biostatistics and Evidence-Based Research, the Parker Institute, Bispebjerg and Frederiksberg Hospital, Copenhagen, Denmark; 9 Institute of Regional Health Research, University of Southern Denmark, Odense, Denmark; 10 Cambridge Institute of Therapeutic Immunology and Infectious Disease, Jeffrey Cheah Biomedical Centre, Cambridge Biomedical Campus, Cambridge, CB 0AW UK; 11 Department of Medicine, University of Cambridge School of Clinical Medicine, Cambridge, CB2 0QQ UK; 12 Life & Medical Sciences (LIMES) Institute, University of Bonn, Bonn, Germany; 13 PRECISE Platform for Single Cell Genomics and Epigenomics, German Center for Neurodegenerative Diseases (DZNE), and University of Bonn, Bonn, Germany

**Keywords:** Intestinal inflammation, Personalized medicine, Biologics, Therapy response, Biomarker

## Abstract

**Background and aims:**

Treatment with tumor necrosis factor α (TNFα) antagonists in IBD patients suffers from primary non-response rates of up to 40%. Biomarkers for early prediction of therapy success are missing. We investigated the dynamics of gene expression and DNA methylation in blood samples of IBD patients treated with the TNF antagonist infliximab and analyzed the predictive potential regarding therapy outcome.

**Methods:**

We performed a longitudinal, blood-based multi-omics study in two prospective IBD patient cohorts receiving first-time infliximab therapy (discovery: 14 patients, replication: 23 patients). Samples were collected at up to 7 time points (from baseline to 14 weeks after therapy induction). RNA-sequencing and genome-wide DNA methylation data were analyzed and correlated with clinical remission at week 14 as a primary endpoint.

**Results:**

We found no consistent *ex ante* predictive signature across the two cohorts. Longitudinally upregulated transcripts in the non-remitter group comprised TH2- and eosinophil-related genes including *ALOX15*, *FCER1A*, and *OLIG2*. Network construction identified transcript modules that were coherently expressed at baseline and in non-remitting patients but were disrupted at early time points in remitting patients. These modules reflected processes such as interferon signaling, erythropoiesis, and platelet aggregation. DNA methylation analysis identified remission-specific temporal changes, which partially overlapped with transcriptomic signals. Machine learning approaches identified features from differentially expressed genes cis-linked to DNA methylation changes at week 2 as a robust predictor of therapy outcome at week 14, which was validated in a publicly available dataset of 20 infliximab-treated CD patients.

**Conclusions:**

Integrative multi-omics analysis reveals early shifts of gene expression and DNA methylation as predictors for efficient response to anti-TNF treatment. Lack of such signatures might be used to identify patients with IBD unlikely to benefit from TNF antagonists at an early time point.

**Supplementary Information:**

The online version contains supplementary material available at 10.1186/s13073-022-01112-z.

## Background

Blockade of the cytokine tumor necrosis factor (TNF) has evolved as a therapeutic concept that is a mainstay for IBD therapy more than 20 years after its first use in patients [1–4]. Primary non-response rates of TNF antagonists vary between 10 and 40% in real cohorts and up to 46% of the patients experience a secondary loss of response in the first 12 months [1, 5, 6]. Moreover, anti-TNF therapy can have several adverse effects in patients, e.g., lupus-like symptoms or increased susceptibility to infections and cancer. Hence, there is an imperative need to find biomarkers that could predict therapy response before or at initial stages of the therapy to reduce unnecessary costs and complications. Several previous studies have defined molecular predictors of therapy response to TNF antagonists [7–18]. However, most of them have focused on investigating pre-selected sets of cellular or transcriptomic signatures at a single time point before the onset of therapy and thereby neglect the dynamic molecular changes that arise upon drug exposure.

We here hypothesized that dynamic changes of genomic network states associated with an adequate response to anti-TNF therapy cannot be reliably understood from a single sampling point. Analyzing early changes after the first drug administration rather than the ex ante molecular signatures could provide mechanistic insights into rewiring of regulatory networks involved in response vs. non-response and could potentially reveal robust predictors of the effectiveness. Longitudinal omics-driven analyses from peripheral blood have proven to be a powerful tool to study trajectories of immune-mediated diseases and can be used to infer markers, which predict clinical outcome [19–21].

We analyzed whole blood samples from IBD patients before and at up to 6 time points after therapy induction by RNA-sequencing and DNA methylation profiling. Using an integrative bioinformatic approach, we identify dynamic biomarker signatures that predict response to TNF antagonist early after therapy initiation. Signatures were replicated in a second independent cohort and selected markers were validated in an additional cohort.

## Methods

### Patient recruitment and study design

Two independent cohorts of IBD patients receiving anti-TNF therapy (discovery and replication cohorts, *n* = 14/23) as well as a cohort of IBD patients receiving vedolizumab as a therapy control (*n* = 17) were recruited for longitudinal biomaterial collection and subsequent RNA and DNA methylome profiling. All patients had clinically active disease (as assessed by clinical indices, endoscopy, and lab parameters) before treatment and received infliximab, adalimumab, or vedolizumab induction therapy following standard medical criteria at the University Hospital. The study design had no influence on therapy decisions. Remission was assessed clinically at 14 weeks based on Harvey-Bradshaw Index (HBI; ≤ 4) for CD and partial Mayo score (≤ 2) for UC patients, respectively. The study was approved by the ethics committee of the Christian-Albrechts-Universität zu Kiel (A 124/14 and AZ 156/03-2/13) and subjects provided written informed consent. Additionally, a publicly available dataset was used as an additional validation cohort [14].

#### Discovery cohort

**Fig. 1:**
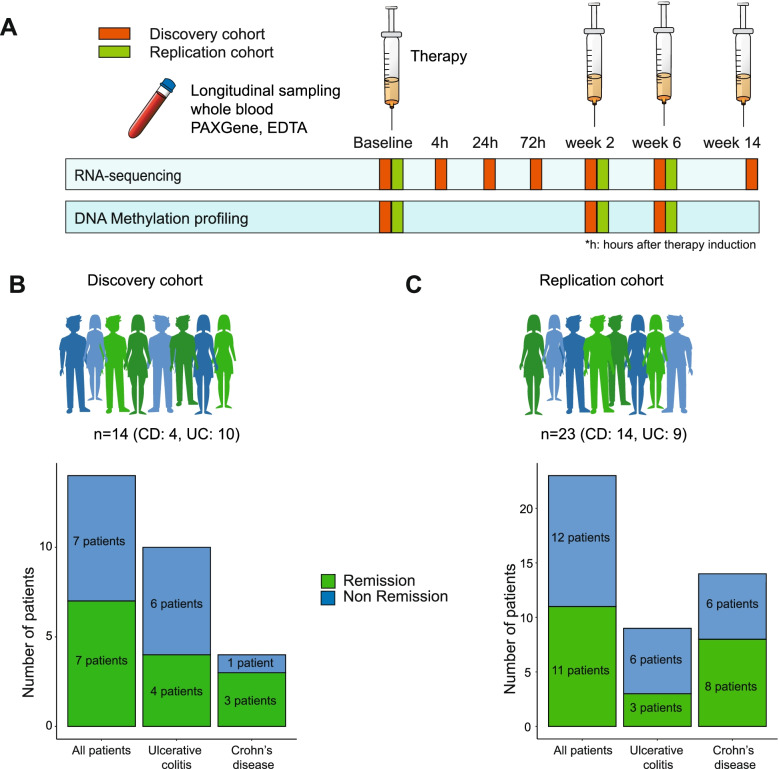
Study design and cohorts. **A** Schematic representation of the study design. **B**, **C** Total number of IBD patients recruited in the discovery (**B**) and replication (**C**) cohorts

The discovery cohort consists of 14 IBD patients (10 UC/4 CD) undergoing first-time treatment with TNF antagonists that were investigated (Fig. 1A, B), 7 of which achieved clinical remission at week 14 (50%). The patient characteristics are described in Table 1. Peripheral blood samples were collected immediately before treatment (baseline); 4, 24, and 72 h; and 2, 6, and 14 weeks after the induction of therapy for RNA sequencing (PAXgene™), while EDTA-stabilized blood from baseline and 2 and 6 weeks after induction was used for genome-wide methylome profiling (Additional file 1: Table S1).

**Table 1:** Clinical characteristics of the discovery cohort. Values represent median ± standard deviation

Discovery cohort	All patients (***n***=14)	Crohn’s disease (***n***=4)		Ulcerative colitis (***n***=10)		Range
Age (y)	38.6 ± 13.6	32.3 ± 14.6		38 ± 11.8		16–62
BMI	26.5 ± 6.8	27 ± 8.6		27 ± 7.1		19.7–38.9
Female sex, *n* (%)	7 (50%)	3 (75%)		4 (40%)		
Time since diagnosis, y	14 ± 11	12 ± 6		16.1 ± 12		6–46
Smokers, *n* (%)	3 (21.4%)	1 (25%)		2 (20%)		
Prednisolone >20mg/day, *n* (%)	2 (14.3%)	1 (25%)		1 (10%)		
Budesonide, *n* (%)	1 (7.1%)	0		1 (10%)		
Thiopurines, *n* (%)	3 (21.4%)	1 (25%)		2 (20%)		
Mesalamine therapy, *n* (%)	12 (85.7%)	2 (50%)		9 (90%)		
Clinical remission at week 14, *n* (%)	7 (50%)	3 (75%)		4 (40%)		
Remitters (R)/non-remitters (NR)		**R**	**NR**	**R**	**NR**	
C-reactive protein, baseline		15.1±16.3	12.0	9.3±9.2	17.1±7.7	
C-reactive protein, week 2		4.1±3.5	3.6	2.6±3.1	13.8±12.2	
C-reactive protein, week 6		5.4±3.5	5.4	7.2±13.0	6.4±7.8	
C-reactive protein, week 14		8.6±3.7	4.8	3.0±8.0	8.3±12.6	
HBI/pMAYO score, baseline		7±1	12	5.5±2.4	6±1.1	
HBI/pMAYO score, week 2		6±0.6	12	3±2.4	5±1.8	
HBI/pMAYO score, week 6		3±2.1	12	2.5±1.1	6±2.2	
HBI/pMAYO score, week 14		1±2.1	12	0.75±0.9	6±1.2	

#### Replication cohort

A second independent replication cohort comprising 23 subsequent IBD patients (9 UC/14 CD; Fig. 1C, Table 2) treated with a TNF antagonist (22 infliximab, 1 adalimumab) for their first time was used to replicate results from the discovery cohort. In this replication cohort, remission at week 14 was achieved in 11 patients (48%). Patient characteristics of this cohort are detailed in Table 2. Blood in PAXgene™ and in EDTA-stabilized containers was collected before (baseline) and 2 and 6 weeks after therapy induction and was used for RNA and methylome profiling (Additional file 1: Table S1).

**Table 2:** Clinical characteristics of the replication cohort. Values represent median ± standard deviation

Replication cohort	All patients (***n***=23)	Crohn’s disease (***n***=14)		Ulcerative colitis (***n***=9)		Range
Age (y)	37.1 ± 11.9	35.0 ± 12.1		40.8 ± 11.5		18–54
BMI	23.9 ± 4.2	23.9 ± 4.3		24.0 ± 4.4		18.7–32.1
Female sex, *n* (%)	11 (47.8%)	6 (42.8%)		5 (55.6%)		
Time since diagnosis, y	5.0 ± 7	5.8 ± 9		3.8 ± 5		0–28
Smokers, *n* (%)	7 (30.4%)	7 (50%)		0		
Prednisolone >20mg/day, *n* (%)	6 (26.1%)	4 (28.6%)		2 (22.2%)		
Budesonide, *n* (%)	5 (21.7%)	4 (28.6%)		1 (11.1%)		
Thiopurines, *n* (%)	6 (26.1%)	3 (21.4%)		3 (33.3%)		
Mesalamine therapy, *n* (%)	9 (39.1%)	4 (28.6%)		5 (55.6%)		
Clinical remission at week 14, *n* (%)		8 (57.1%)		3 (33.3%)		
Remitters (R)/non-remitters (NR)		**R**	**NR**	**R**	**NR**	
C-reactive protein, baseline		5.1±8.25	7.7±19.7	2.4±1.3	8.7±4.5	
C-reactive protein, week 2		1.1±2.1	3.3±4.7	1±0.1	3.3±4.7	
C-reactive protein, week 6		2.7±3.7	4.2±3.8	2.2±1.9	3.6±4.4	
C-reactive protein, week 14		3±4.2	5±3.7	15.5±20.6	4.8±11.9	
HBI/pMAYO score, baseline		9±4.1	7.5±2.6	5±0	6±1.6	
HBI/pMAYO score, week 2		5±3.0	5.5±0.8	4.5±0.7	5±2.3	
HBI/pMAYO score, week 6		2±2.4	6±1.4	2±1.4	5±1.1	
HBI/pMAYO score, week 14		0.5±1.5	5±0.9	2±0	6±0.5	

#### Validation cohort

For validation of predictive biomarkers identified from the discovery and replication cohorts, published microarray data from 20 CD patients with peripheral blood expression data from baseline and 2 weeks after infliximab therapy (validation cohort) [14] was employed.

#### Vedolizumab cohort

Peripheral blood samples from another cohort of 17 IBD patients (10 UC/7 CD) undergoing treatment with an anti-α4β7 integrin antibody (vedolizumab) were used for contrasting molecular signatures specific to treatment with TNF antagonists. This cohort was recruited in parallel with the discovery cohort with the same study design and characteristics have already been described in another study by Zeissig et al. [22]. In the previous study, however, only biopsy samples were analyzed, while we utilized blood collected in PAXgene™ tubes at baseline; 4, 24, and 72 h; 2, 6, and 14 weeks for RNA profiling (Additional file 1: Table S1). Nine out of 17 IBD patients (53%) treated with vedolizumab achieved clinical remission at week 14.

### RNA sequencing and analysis

Blood (2.5 mL) was taken from each patient into a PAXgene™ Blood RNA Tube, containing a patented RNA stabilizer reagent composition. RNA was isolated in QIAGEN’s QIAcube using the PAXgene Blood miRNA Kit from QIAGEN PreAnalytiX. RNA-sequencing libraries were prepared according to the Illumina TruSeq® messenger RNA (mRNA) sequencing protocol (TruSeq® RNA Seq Library Prep Kit v2) and sequenced on an Illumina HiSeq 4000 (2 × 75 bp) [23].

An in-house RNA-seq pipeline was used to map and align the sequenced data [24]. Adapters and low-quality bases from the RNA-seq reads were removed using Trim Galore (version 0.4.4) [25] and reads shorter than 35 bp after trimming were discarded. The filtered reads were mapped to the human genome (GRCh38, gencode version 25) using a STAR aligner (version 2.5.2b) [26]. Expression counts were estimated using featureCounts (version 1.5.2) [27] and normalized across samples using the DESeq normalization method [28].

Differentially expressed genes (DEGs) were identified using two different approaches: pairwise and longitudinal. Both approaches were applied to the transcriptomic data for remission and non-remission patients separately. In the pairwise analysis, gene expression at each time point after therapy was compared against that at baseline using the bioconductor package DESeq2 (version 1.20.0) [28]. Longitudinal analysis was performed by applying the case-only analysis from the bioconductor package ImpulseDE2 (version 1.4.0) [29]. Genes with FDR adjusted *p*-value of 0.05 in both analyses were regarded as differentially expressed.

### Gene co-expression analysis

Modules of co-expressed genes were identified for the samples at baseline using the WGCNA package for R [30]. Genes that were differentially expressed only in remission patients were used to generate the gene co-expression modules. First, pairwise gene correlations were calculated based on the log-transformed normalized expression counts across all samples. A signed adjacency matrix was constructed by applying a soft threshold function with a power of 16, which was the minimum power for which the scale-free fit was greater than 0.9. The adjacency matrix was used to construct a gene tree by hierarchal clustering. Genes were then split into modules based on the gene tree by using the function cuttreeDynamic with the minimum module size set to 15. Modules that were closely related were then merged using the function mergeCloseModules with parameter cutHeight set to 0.2. The preservation of the modules identified at baseline was tested at weeks 2 and 6 using WGCNA in remission and non-remission samples and quantified using the *Z*_summary_ score [31, 32]. To associate gene co-expression modules with the clinical parameters and to visualize the expression profile of the genes in a module, module eigengene values for the samples were calculated. Spearman’s rank correlation coefficients were calculated between module eigengenes and clinical parameters, such as HBI score, partial Mayo score, C-reactive protein (CRP), fecal calprotectin, leukocytes, interleukin-6 (IL-6), and disease status at week 14.

### DNA methylation profiling and analysis

Infinium MethylationEPIC BeadChip (Illumina) was used to measure DNAm levels from EDTA blood samples according to the manufacturer’s protocol [33]. DNA methylation data was analyzed using the Bioconductor package RnBeads (version 1.12.1) [34]. Sites that overlapped with SNPs and had unreliable measurements were filtered resulting in the removal of 17,371 sites and 20,876 probes. A total of 2976 context-specific probes, 18,837 probes on the sex chromosomes, and 10 probes with missing values were also removed. In total, 42,699 out of 866,895 probes were filtered. The signal intensity values were normalized using the dasen method. Differentially methylated positions (DMPs) and regions (DMRs) between baseline and week 2 and baseline and week 6 samples from the remitting and non-remitting patients were identified using the automatically selected rank cutoff of RnBeads. The chi-square test was used to calculate the statistical significance of the over- or under-representation of DMPs in known gene, promoter, and enhancer regions.

### DNA methylation-transcriptome integrated analysis

For the integrated analysis of gene expression with DNA methylation, DMPs located 5000 bp upstream and downstream of the transcription start sites of DEGs were identified and Spearman’s rank correlation coefficient between normalized expression of each DEG and methylation intensity of its corresponding DMPs were calculated as described [21]. To test the statistical significance of the correlations, a false discovery rate (FDR) using a permutation approach was calculated.

### Functional enrichment analysis

All gene ontology enrichment analyses were conducted using the Bioconductor package topGO (version 2.32.0) [35]. In the topGO analysis, the Fisher.elim *p*-value, calculated using the weight algorithm of 0.05 was used as significance threshold. Transcription factor binding sites (TFBS) enriched in DMPs and DMRs were identified by conducting enrichment analysis using the Bioconductor package LOLA (version 1.14.0) [36].

### Feature selection and machine learning

Feature selection was performed using a random forest approach implemented in the ranger package (version 0.12.1) of R [37]. Prediction models were built using the caret package of R based on the random forest approach [38]. The data from discovery and replication cohorts were used as input and AUC and ROC curves under 10-fold cross-validation were used to access the accuracy of the models.

## Results

### Cohorts

To delineate the molecular signatures of therapy response to TNF antagonists, we performed a longitudinal analysis of the blood transcriptome and epigenome of two individual clinical cohorts of IBD patients in a case-only design (Fig. 1A). For the discovery cohort (Fig. 1B), whole blood samples were collected from 14 IBD patients (10 UC/4 CD) (Table 1). Seventeen IBD patients (10 UC/7 CD), who received first-time therapy with vedolizumab [22, 23], a monoclonal antibody directed against α4β7 integrin, were used as treatment controls. For the discovery cohort, we employed RNA-sequencing on samples collected at all time points (baseline; 4, 24, and 72 h; 2, 6, and 14 weeks after the first infusion) [23]. Genome-wide DNA methylation profiling was done on baseline, week 2, and week 6 samples [33]. Therapy outcome (primary endpoint) was defined based on the achievement of clinical remission at week 14, as assessed by clinical disease activity markers (Harvey-Bradshaw Index ≤ 4 for CD; partial Mayo score ≤ 2 for UC). Of the 14 IBD patients treated with infliximab, 7 achieved clinical remission at week 14 (50%) and 9 out of 17 IBD patients (53%) treated with vedolizumab achieved clinical remission at week 14. Results from the discovery cohort were replicated in a second independent replication cohort comprising 23 subsequent IBD patients (9 UC/14 CD; Fig. 1C, Table 2) treated with a TNF antagonist (22 infliximab, 1 adalimumab) [23]. Here, samples were taken at baseline, week 2, and week 6. In this cohort, remission at week 14 was achieved in 11 patients (48%).

### Dynamics of transcriptomic changes upon TNF antagonist exposure

**Fig. 2:**
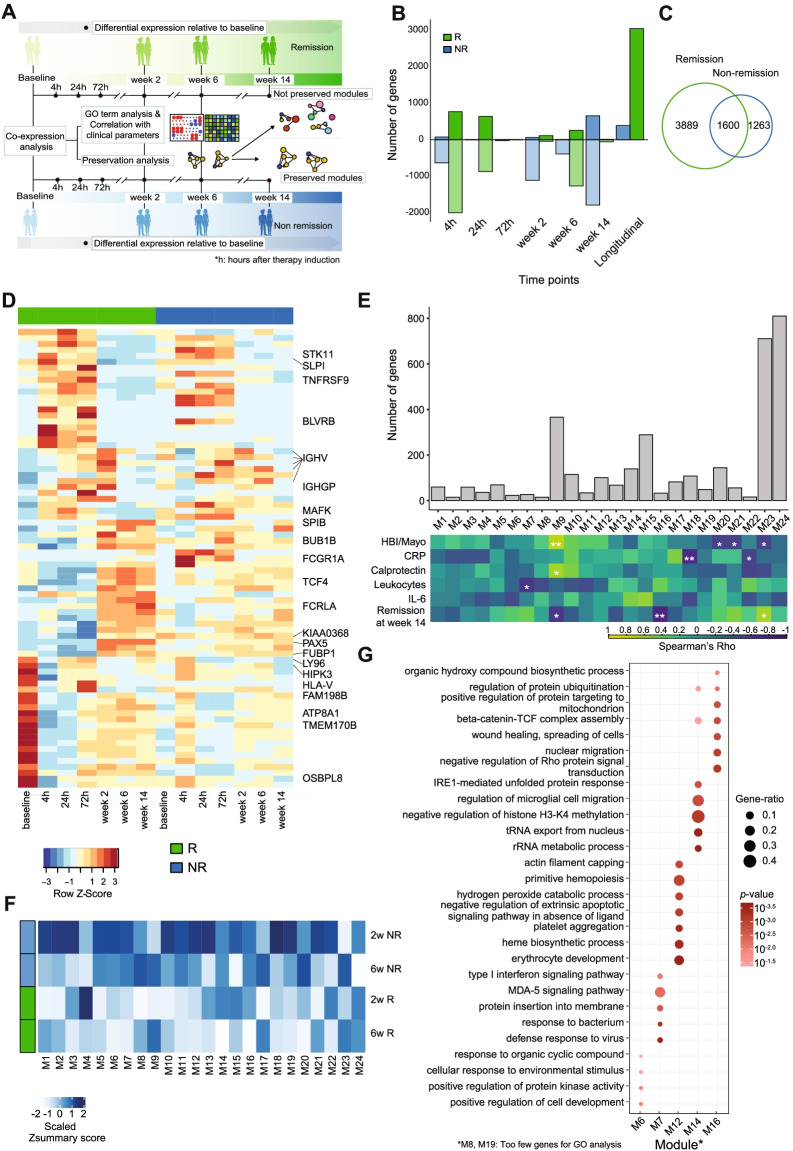
Dynamic changes in transcription in response to therapy induction and remission. **A** Schematic workflow. **B** Number of upregulated (dark) and downregulated (light) genes in remission (green) and non-remission (blue) patients at each time point after therapy induction obtained from the pairwise analysis and number of transiently differentially expressed genes obtained from the longitudinal analysis of the discovery cohort. Negative numbers are used to show the number of downregulated genes. **C** Venn diagram showing the number of DEGs in remission and non-remission patients from pairwise and longitudinal analysis combined. **D** Heatmap of top DEGs in remission patients from pairwise and longitudinal analysis, showing scaled mean expression counts at each time point in remission and non-remission samples. Selected immune-relevant transcripts are labeled by gene name. **E** Bar plot showing the number of genes in each co-expression module along with a correlation heatmap showing Spearman’s rank correlation coefficients between gene co-expression modules (columns) and clinical parameters (rows). *p-value < 0.05, **p-value < 0.01, and ***p-value < 0.001 in Spearman’s correlation. Color intensity corresponds to the correlation coefficient. **F** Heatmap showing *Z*_summary_ scores of baseline co-expression modules in remission and non-remission samples at weeks 2 and 6. **G** GO terms enriched in differentially preserved co-expression modules between remission and non-remission. Dot size is proportional to the gene ratio and color corresponds to the *p*-value of enrichment

To investigate the dynamics of transcriptional responses of IBD patients after therapeutic exposure to a TNF antagonist, we analyzed longitudinal whole blood transcriptomic data before (baseline) and at up to 6 time points after the introduction of infliximab therapy in the discovery cohort (Fig. 1A). We first compared the transcriptional signatures between remitters and non-remitters at baseline to identify any prior signature of therapy response. Through principal component analysis (PCA), we observed a suggestive *ex ante* separation between patients achieving remission and non-remission at week 14 along the PC2 axis (Spearman’s rho = 0.58, *p*-value = 0.04; Additional file 2: Fig. S1A). However, after taking the diagnosis into account, we observed that separation on PC2 mainly reflected the difference between CD and UC patients (partial correlation coefficient with diagnosis = 0.65, *p*-value = 0.02, partial correlation coefficient with disease status at week 14 = 0.46, *p*-value = 0.1; Additional file 2: Fig. S1A). The first two principal components did not associate with age, gender, or concomitant medication usage (Additional file 2: Fig. S1B, C). Differential expression analysis, after taking diagnosis as a covariate, further identified 387 genes that were nominally differentially expressed between remitters and non-remitters at baseline (Additional file 2: Fig. S1D). We next performed pairwise differential expression analyses between baseline and each of the time points after therapy initiation in the discovery cohort in remitters and non-remitters separately (Fig. 2A). Overall, treatment with infliximab led to profound alterations in the blood transcriptome within the first 24 h after drug exposure with transcript levels of most differentially expressed genes (DEGs) being downregulated (Fig. 2B, D, Additional file 1: Table S2, S3). Furthermore, we observed that patients who attained remission showed overall higher numbers of DEGs, pointing towards molecular response trajectories starting as early as 4 h after therapy exposure (Fig. 2B). The inter-individual heterogeneity, quantified by the variance in gene expression, was also significantly higher in non-remitters compared to remitters at all time points except week 14 (data not shown). ImpulseDE2 was employed to construct a continuous temporal model of gene expression [29] over time. We identified 3043 DEGs with significant impulse-like progression patterns across time points in remitting patients, whereas only 389 DEGs were identified in non-remitting patients (Fig. 2B). Pairwise and longitudinal analyses were combined in remission and non-remission patients (Additional file 1: Table S2, S3). A total of 1600 genes were shared between the groups (Fig. 2C).

Gene ontology enrichment analysis on DEGs at each time point identified complex inflammatory processes from downregulated gene sets from week 2 onwards in both remitters and non-remitters. “Positive regulation of NF-kB transcription factor activity” and “toll-like receptor signaling pathway” were among the uniquely enriched terms in downregulated genes at week 2, 6, or 14 in patients achieving remission at week 14 (Additional file 2: Fig. S2A). Terms such as “positive regulation of leukocyte degranulation” and “integrin-mediated signaling pathway” were uniquely enriched in downregulated genes in non-remission patients from 2 to 14 weeks after therapy induction (Additional file 2: Fig. S2B). Interestingly, the small set of upregulated transcripts in the non-remitter group comprised TH2- and eosinophil-related genes including *ALOX15*, *FCER1A*, and *OLIG2* (Additional file 1: Table S3). These observations indicated that modulation of immune network states by antagonizing TNF in blood is complex and that even in patients who did not achieve remission at week 14, several pro-inflammatory processes are dampened in this compartment. Since classical rank-based gene expression analysis did not clearly distinguish between the therapy outcomes, we applied higher-order gene expression regulation analysis to find distinct features associated with therapy response.

### Dysregulation of co-expression networks during the induction of remission

To further annotate and condense functional groups of genes, which are linked to effective anti-TNF therapy, we next analyzed gene co-expression networks using weighted gene co-expression network analysis (WGCNA) [30]. Co-expression analysis follows the assumption that clusters of genes with similar expression patterns (so-called modules) are likely to share regulatory inputs and biological function or are derived from a specific cell type in complex tissue samples [21]. We hypothesized that co-expression patterns of differentially expressed genes could change over the course of a targeted therapy, which — although not completely unbiased — may allow a focused view on pathways that are disrupted during the induction of remission. We therefore constructed focused co-expression networks (as described in [39, 40]) using all DEGs separating remitters from non-remitters (pairwise and longitudinal combined; 3889 genes), which were identified in the previous analysis step. We started from baseline samples and compared the preservation of modules at week 2 and week 6 between patient groups, stratified according to the respective therapeutic outcome (Fig. 2A). These time points were chosen because they reflect the intermediate state between active disease at baseline and primary endpoint at which the therapy outcome was defined. This approach resulted in a total of 24 co-expression modules (Fig. 2E, M1–M24). We calculated the respective eigengene values, which represent a single expression profile for all genes within a module and correlated these values to respective clinical parameters (Fig. 2E) as well as to computationally inferred cell type proportions (Additional file 2: Fig. S2C) [41].

We next analyzed the preservation of modules at week 2 and week 6, stratified according to the respective therapeutic outcome at week 14 (Fig. 2A). We applied *Z*_*summary*_ statistics as a measure of module preservation [32]. M6, M7, M8, M14, and M16 modules were moderately preserved in non-remission (2 < *Z*_*summary*_ < 10) while not preserved in remission (*Z*_*summary*_ < 2) at one or both time points (Fig. 2F, G, Additional file 2: Fig. S2D). Modules M12 and M19, on the other hand, were highly preserved in non-remission (*Z*_*summary*_ > 10) and significantly less preserved in remission (2 < *Z*_*summary*_ < 10) (Fig. 2F, Additional File 2: Fig. S2D). Genes in the differentially preserved modules were involved in diverse biological processes, e.g., type I interferon signaling pathway, MDA-5 signaling pathway and interleukin-1 beta secretion in module M7 and platelet aggregation, erythrocyte development, and ROS signaling in M12 (Fig. 2G). Altogether, using co-expression analysis, we identified salient transcriptional modules that change during the therapy, specifically in patients that achieve remission at week 14.

### Unique molecular signatures induced by TNF inhibition

**Fig. 3:**
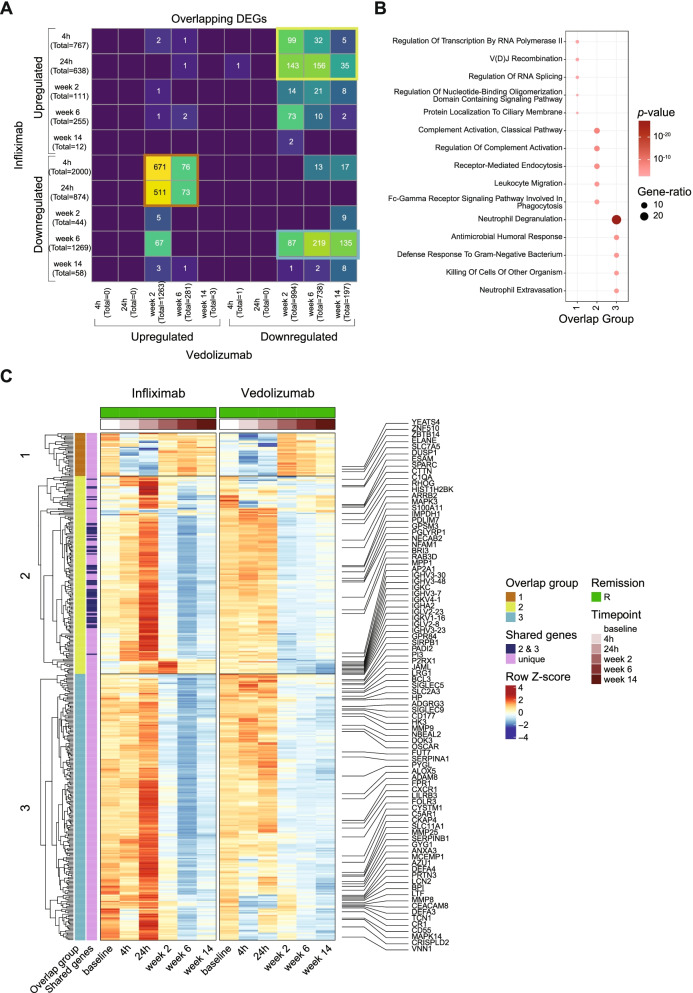
Comparison of transcriptomic changes between infliximab and vedolizumab patients. **A** Cross-tabulation of genes differentially expressed in patients treated with infliximab (rows) and vedolizumab (columns) that achieved remission after 14 weeks of the respective therapy induction. The three groups of overlapping genes are highlighted in orange (group 1), green (group 2), and blue (group 3). **B** GO terms enriched in genes belonging to the three overlap groups. Dot size is proportional to the gene ratio and color corresponds to the *p*-value of enrichment. The top five GO terms in each group are visualized. **C** Heatmap showing average scaled mean expression counts at each time point of selected genes in the three overlap groups

To identify the unique molecular signatures induced by treatment with infliximab, we compared the differentially expressed genes at each time point in IBD patients treated with infliximab to that in IBD patients treated with vedolizumab who attained remission after 14 weeks of respective therapy initiation. We found that the transcriptional dysregulation observed within the first 24 h in infliximab-treated patients was not shared in vedolizumab-treated patients (Fig. 3A). We observed three large groups of overlapping genes between the two treatments: (1) downregulated genes at early time points (4h, 24h) in infliximab-treated patients that were upregulated at later time points (weeks 2 and 6) in vedolizumab-treated patients, (2) upregulated genes at early time points (4h, 24h) in infliximab-treated patients that were downregulated at later time points (weeks 2, 6, and 14) in vedolizumab-treated patients, and (3) shared downregulated genes in both treatments at later time points (Fig. 3A). The first two groups that showed contrasting expression patterns between the two treatments could describe the unique mechanism of action of infliximab while the third group could represent the overall signature of healing and decline in inflammation. Group 1 genes were enriched in processes related to transcription and splicing as well as V(D) J recombination and mainly consisted of genes that were highly expressed at baseline in infliximab-treated patients (Fig. 3B, C). Group 2 genes were related to complement activation, leukocyte migration, and endocytosis and showed a strong upregulation at 24h specifically in patients remitting after 14 weeks of infliximab treatment (Fig. 3B, C). The last group (group 3) had a similar expression pattern at later time points between the two treatments and consisted of genes related to neutrophil degranulation and humoral response (Fig. 3B, C). Taken together, we identified a transcript signature that was regulated in a contrasting manner between treatments that target TNF and α4β7 integrin as well as genes that indicate a systemic reduction in inflammation that were shared between the two treatments.

### Dynamic changes in genome-wide methylation

**Fig. 4:**
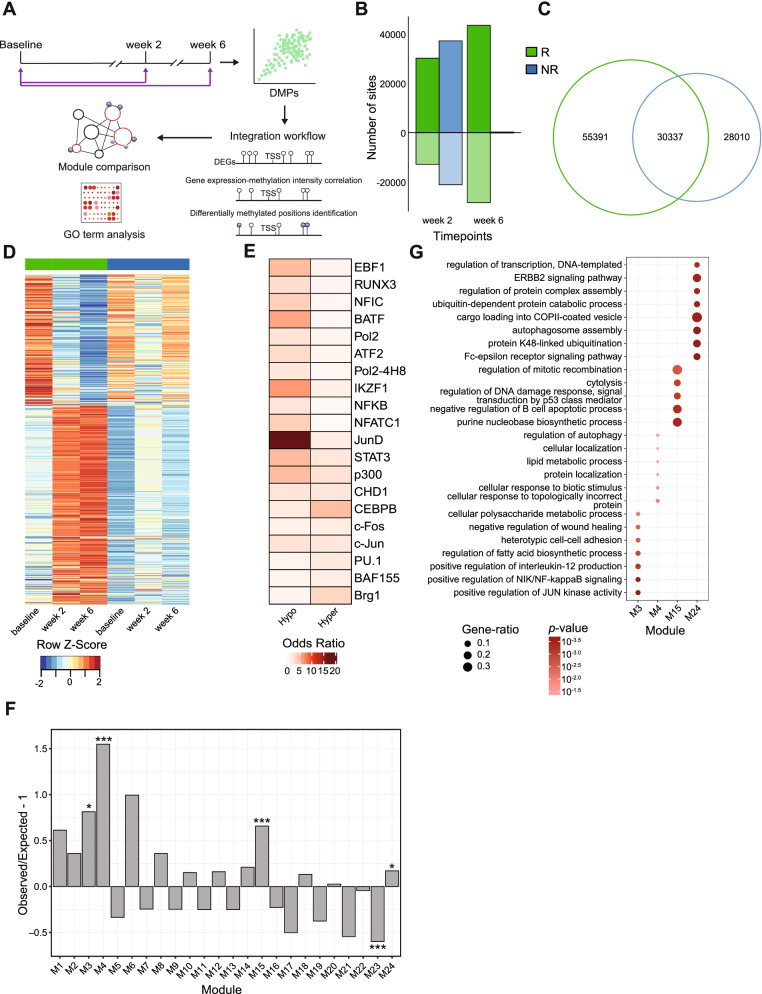
DNA methylation analysis and integration of omics layers. **A** Schematic workflow. **B** Number of hypermethylated (dark) and hypomethylated (light) positions in remission (green) and non-remission (blue) patients at each time point after therapy induction obtained from the pairwise analysis of the discovery cohort. Negative numbers are used to show the number of hypomethylated positions. **C** Venn diagram showing the number of DMPs in remission and non-remission patients. **D** Heatmap of DMPs, which are correlated with DEGs, showing scaled mean methylation intensities at each time point in remission and non-remission samples. **E** Heatmap showing significant enrichment, quantified by odds ratio, of transcription factor binding sites (TFBS) in DMPs that are correlated with DEGs. Selected top TFs are visualized. **F** Over-representation and under-representation of DNAm-linked DEGs in co-expression modules. The over-/under-representation is quantified as the ratio of the observed and expected number of correlated genes present in each module under the chi-square distribution. **G** GO terms enriched in DNAm-linked co-expression modules. Dot size is proportional to the gene ratio and color corresponds to the *p*-value of enrichment

DNA methylation (DNAm) is an important epigenetic mechanism for long-term regulation of gene expression, which has been shown to be involved in the etiopathogenesis of IBD [42–44]. We thus analyzed DNAm signatures by bead arrays covering > 850,000 CpG sites across the entire genome before and 2 and 6 weeks after the administration of infliximab therapy (Fig. 4A). We used a pairwise approach to interrogate differentially methylated sites and regions between baseline and week 2 samples and baseline and week 6 samples [34]. We identified a total of 85,728 and 58,347 differentially methylated positions (DMPs) in remitters and non-remitters, respectively (Fig. 4B, C, Additional file 2: Fig. S3B, S3C). In the samples of patients achieving remission at week 14, a preponderance of hypermethylated DMPs was observed, constituting around 70% (30,132) at 2 weeks and 60% (43,478) of the DMPs at 6 weeks (Fig. 4B). Cellular deconvolution analysis [34] identified that major parts of the observed DNAm signatures originated from granulocytes, B cells, CD4^+^ T cells, and monocytes, similar to the transcriptional signatures (Additional file 2: Fig. S3A). The inferred granulocyte proportions in blood significantly decreased across time points only in remitting patients (linear mixed model ANOVA *p*-value = 0.043).

In total, 357 differentially methylated regions (DMRs) such as promoters, genes, CpG island, and enhancers were observed in remitters and 1163 DMRs in non-remitters (Additional file 2: Fig. S3D, S3E). The majority of the DMRs belonged to enhancer regions (348 in remission and 1147 in non-remission), consistent with the distribution of the DMPs (Additional file 2: Fig. S3B, S3D, S3E). These DMRs overlapped with binding sites for several transcription factors including IRF4, BATF, MEF2C, and MEF2A for hypermethylated regions and CEBPD and STAT3 for hypomethylated regions (Additional file 2: Fig. S3F, S3G). Interestingly, most DMPs and DMRs in non-remitting patients were transiently observed only at week 2, while many DMPs were stably regulated at week 2 and at week 6 in remitting patients (Additional file 2: Fig. S3C).

### Analysis of DNAm-linked transcriptomic changes

To link DNA methylation changes to gene expression *in cis*, we performed an integrative analysis using a hierarchical approach [21], which identified DMPs located within 5kb upstream or downstream of the transcription start site of each DEG. We then calculated the correlation between gene expression of each DEG and methylation intensity of the corresponding DMPs (Fig. 4A). Out of a total of 85,728 remission-associated DMPs (DMPs at week 2 and week 6 combined), 5459 were in a 5-kb vicinity of at least one DEG. In total, 1253 DMP-DEG pairs (representing 763 genes) were significantly correlated. 65.9% of cases followed a canonical negative correlation (i.e., high methylation-low expression) (Additional file 2: Fig. S4A, Additional file 1: Table S4). DMPs that correlated with the DEGs showed a persistent hypo- or hypermethylation after therapy initiation in patients achieving remission at week 14 and overlapped with binding sites for transcription factors BATF, NF-κB, JunD, STAT3, and CEBPB among others (Fig. 4D, E, Additional file 2: Fig. S4C). This pattern was, however, completely absent in non-remitting patients, supporting our hypothesis of lack of long-term epigenetic changes in patients failing anti-TNF therapy (Fig. 4D, Additional file 2: Fig. S4D). We also investigated the representation of DEG-DMP pairs in the previously defined co-expression modules. DNAm-linked expression changes were significantly overrepresented in modules M3, M4, M15, and M24 (Fig. 4F, Additional file 2: Fig. S4B). These modules were correlated with the inferred proportions of neutrophils, T cells, and NK cells in whole blood (Additional file 2: Fig. S2C). As none of these modules was found to be disrupted by anti-TNF in the prior preservation analysis, this pointed to a rather stable association of identified DMPs and DEGs, possibly reflecting cell types or general inflammatory principles, such as neutrophil proportions (Fig. 4G, Additional file 2: Fig. S2C). Taken together, we were able to identify potentially epigenetically controlled transcriptional changes related to therapy response and induction of remission by integrating DNA methylation data with transcriptomic data. Although we cannot exclude that differences in cellular composition contribute to the above-mentioned observations, several immune-related features indicate a potential long-term alteration of cellular states through this epigenetic process.

### Replication of molecular signatures in an independent clinical cohort

We conducted a formal replication using the same profiling methods (RNA-sequencing and DNAm bead array) in an independent prospective cohort of 23 IBD patients starting anti-TNF treatment (Table 2). To rule out any systematic difference between discovery and replication cohorts, we contrasted the baseline transcriptome signatures from both cohorts with transcriptome signature from (i) 20 healthy individuals and (ii) 15 inactive IBD patients (4 UC/11 CD). While principal component analysis (PCA) showed a separation between healthy controls and IBD patients (Additional file 2: Fig. S5A), we did not observe a significant separation between the discovery and replication cohorts confirming the absence of potential larger batch effects between the two cohorts (Additional file 2: Fig. S5A). A large proportion of disease-related DEGs (IBD vs. healthy) was shared between the two IBD cohorts (Additional file 2: Fig. S5B). The transcriptomic variation in the baseline samples of the replication cohort, represented by the first two principal components, was not associated with disease subtype, disease status at week 14, age, gender, or medication usage (Additional file 2: Fig. S5C, S5D, S5E). Despite the similar characteristics and inclusion criteria of the two cohorts (Tables 1 and 2), we observed little overlap between DEGs or DMPs at baseline (remitters vs. non-remitters) pointing to high heterogeneity of responders before treatment initiation (Additional file 2: Fig. S5C, S6A).

**Fig. 5:**
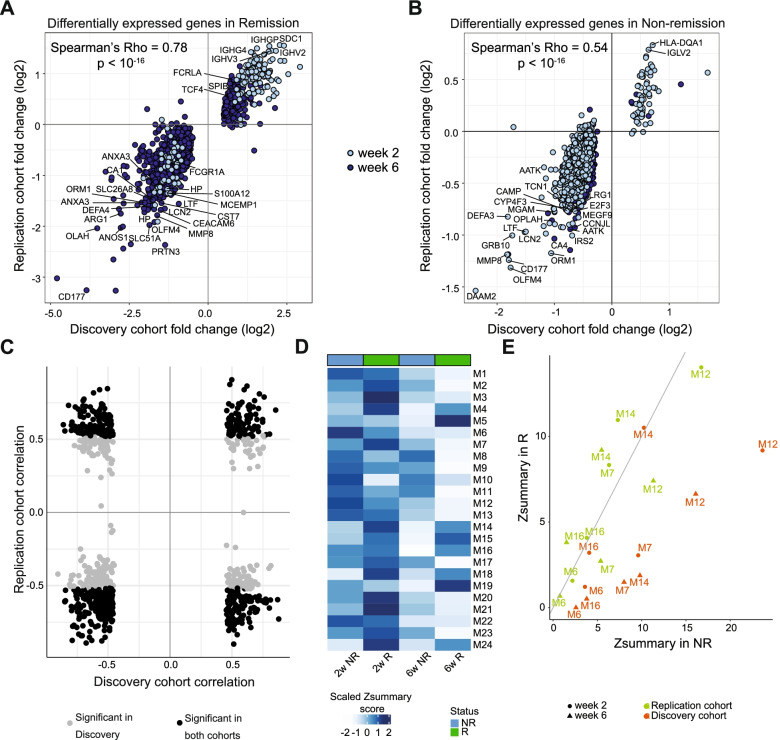
Replication of molecular signatures. **A**, **B** Comparison of log fold change of DEGs in remission (**A**) and non-remission (**B**) patients at weeks 2 (light blue) and 6 (dark blue) between discovery and replication cohorts. **C** Comparison of DEG-DMP correlation between discovery and replication cohorts. Gray dots represent a significant correlation in the discovery cohort while black dots significant correlation in both cohorts. **D** Heatmap showing *Z*_summary_ scores of baseline co-expression modules from the discovery cohort in remission (green) and non-remission (blue) samples at weeks 2 and 6 of the replication cohort. **E** Comparison of *Z*_summary_ scores of differentially preserved modules in discovery cohort between remission and non-remission samples at weeks 2 (circle) and 6 (triangle) in the discovery (orange) and replication (green) cohorts

Next, we aimed to confirm the longitudinal transcriptional and methylation changes observed in the discovery cohort using the replication cohort. We observed that DEGs obtained at week 2 and week 6 in the discovery cohort were similarly regulated in the replication cohort, indicated by a strong correlation between log fold changes with respect to baseline in the two cohorts (Spearman’s rho = 0.78 for remission DEGs, 0.85 for remission-only DEGs, 0.54 for non-remission DEGs and 0.42 for non-remission only DEGs) (Fig. 5A, B, Additional file 2: Fig. S6B, S6C, Additional file 1: Table S5, S6). We repeated the module preservation analysis in the replication cohort and could replicate that modules M7 and M12 were significantly less preserved in remission compared to non-remission at week 6 in this dataset (Fig. 5D, E, Additional file 2: Fig. S6D). To exactly identify the genes that are responsible for loss of module connectivity upon therapy induction, we compared the eigengene-based connectivity measure *kME* or the module membership score, which measures the correlation between the expression of a gene to the consensus expression of the module [30]. In both discovery and replication cohorts, M7 genes such as *RSAD2*, *RIPK2*, *HERC5*, *IFI44*, *CMPK2 SAMD4A*, *MSLN*, *XAF1*, *DDX60*, *RTP4*, and *PARP12* showed the strongest reduction in *kME* in remitting patients (Additional file 2: Fig. S6E). In the M12 module, genes with a loss in connectivity in remitters compared to baseline and non-remitters included *SLC4A1*, *ANK1*, *BLVRB*, *TAL1*, *IFIT1B*, *ACKR1*, *FAM210B*, *TSPAN5*, *E2F5*, and *GATA1* among others in the discovery as well as replication cohort (Additional file 2: Fig. S6F). We also confirmed the correlation between the expression of the DEGs and their nearby methylated sites in the replication cohort. A total of 518 out of the 763 genes were also DNAm-linked in the replication cohort with the direction of correlation preserved in 322 genes (Fig. 5C, Additional file 1: Table S4).

Overall, most of the observed early longitudinal molecular signatures upon anti-TNF induction therapy were reproducibly associated with clinical outcome (endpoint: remission at week 14) in a second cohort of IBD patients, whereas the lack of replication of baseline difference points to a high heterogeneity of prior immune network states, at least in peripheral blood.

### Comparison of IBD subtypes

Due to the small sample size of the discovery cohort, we did not perform data analysis for the IBD subphenotypes separately in each cohort. However, by combining discovery and replication cohorts, we could attain enough statistical power to analyze CD and UC samples separately. Since, the replication cohort was sampled only at baseline, week 2, and week 6, we performed the pooled analysis at only these time points. At baseline, the differential expression analysis between remitters and non-remitters identified no significant differentially expressed genes in CD patients, while 1 DEG (*IGHV1*) was observed in the UC patients.

In the longitudinal pairwise analysis, we observed a higher number of DEGs in CD patients compared to the UC patients (Additional file 2: Fig. S7A). Comparing these results with the DEGs obtained from the analysis of IBD samples of the discovery cohort, we observed that almost all DEGs identified in UC patients who attained remission after 14 weeks were already contained in the IBD analysis whereas the pooled analysis resulted in the identification of many DEGs that were unique to CD (Additional file 2: Fig. S7B). To identify the molecular pathways involved in therapy response that differ between CD and UC patients, we performed gene ontology enrichment analysis on the DEGs unique to CD and the DEGs that were shared between CD and UC. While downregulated genes were enriched in biological processes related to general inflammatory signaling in both diseases, analysis of upregulated genes showed that T cell-specific terms (e.g., regulation of T cell differentiation: *GATA3*, *LAG3*, and *TCF7*) were increased only in CD patients (Additional file 2: Fig. S7C, S7D). TH2/eosinophil signature genes (*ALOX15*, *FECR1A*, and *OLIG2*), identified in the non-remitter group in the IBD analysis, were upregulated in both CD and UC individually as well. Overall, disease-specific analysis recapitulated the patterns observed in IBD analysis, but also revealed certain processes unique to CD.

### Prediction of remission using early molecular changes

**Fig. 6:**
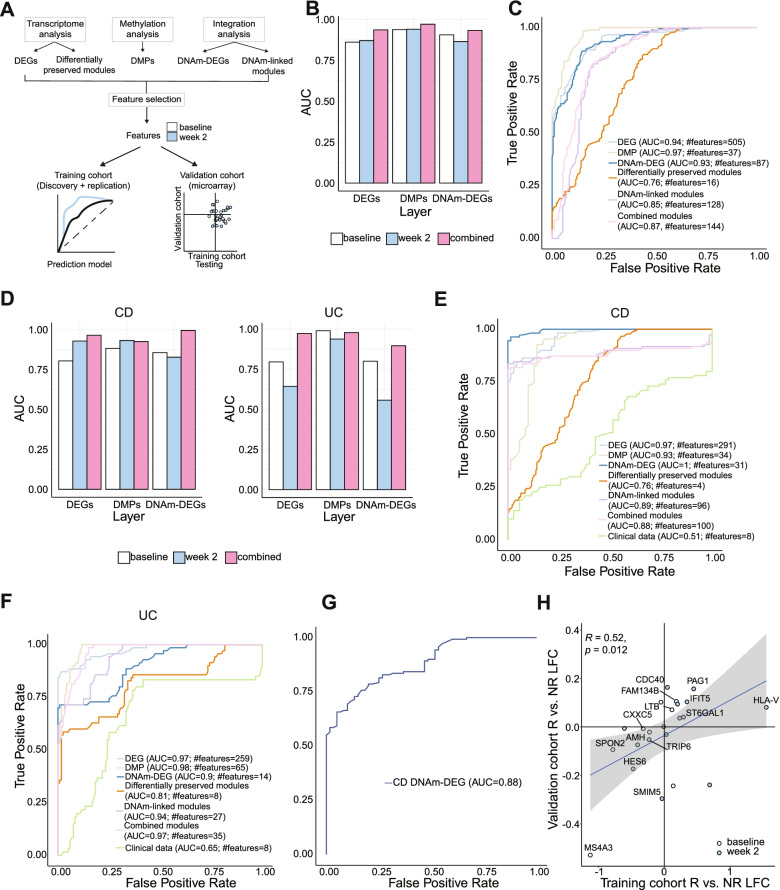
Feature selection and validation of molecular signatures. **A** Schematic workflow. **B**, **D** Comparison of AUC values of the ROC curves of prediction models constructed using selected baseline (white), week 2 (blue), and combined (pink) features from DEGs, DMPs, and DNAm-DEGs using a random forest approach in IBD (**B**), CD, and UC (**D**) samples from the training cohort. **C**, **E**, **F** ROC curves of prediction models constructed using selected features (baseline and week 2 combined) from DEGs, DMPs, DNAm-DEGs, differentially preserved, DNAm-linked, combined modules, and clinical parameters using a random forest approach in IBD (**C**), CD (**E**), and UC (**F**) samples from the training cohort. **G** ROC curve of prediction model constructed using selected features from DNAm-DEGs in the validation cohort. **H** Comparison of log fold change between remitters and non-remitters at baseline (white) and week 2 (blue) (left) between training cohort and validation cohorts

Next, we tested the ability of each layer of molecular information at early time points (baseline vs. week 2) to formally predict therapy response at week 14. For this analysis, we combined the data from discovery and replication cohorts to increase the power of the initial analysis and then validated the results in a publicly available data set [14]. To compare the predictive potential of different individual and combined omics data layers, we performed feature selection and built prediction models on molecular sets derived from transcriptomic analysis (DEGs and differentially preserved modules), methylation analysis (DMPs), and integration analysis (DNAm-DEGs and DNAm-linked modules) (Fig. 6A) using a random forest model with 10-fold cross-validation. This approach showed that models performed better when features from baseline and week 2 were combined (Fig. 6B, D). Prediction models built on CD and UC samples separately performed better than the ones using the combined set of IBD samples (Fig. 6C, E, F). As a control, we also constructed prediction models based on the clinical data of the patients which included CRP, IL-6, serum levels of tryptophan [45], and disease activity scores at baseline and week 2 and compared them to the models from the molecular datasets. Age and gender were not included here since these are stable parameters and there was no significant association observed between these factors and the therapy outcome (CD: age Wilcoxon test *p*-value = 0.26, gender chi-square test *p*-value = 0.26, UC: age Wilcoxon test *p*-value = 0.07, gender chi-square test *p*-value = 0.26). Prediction from models using the clinical data performed worse than the ones built on the molecular datasets (Fig. 6E, F). The model using the selected features from the integration of DNA methylation and gene expression (DNAm-DEGs) was the best performing model for CD with 31 features (AUC=1) (Fig. 6E). In contrast, the models based on molecular signatures from individual omics layers (DEGs: AUC=0.97, #features=259, and DMPs: AUC=0.98, #features=65) outperformed the one based on DNAm-DEGs (AUC=0.9, #features=14) for UC samples (Fig. 6F).

As overfitting is an inherent challenge of machine learning using a single cohort approach even when using cross-validation, we next tested the prediction model built on the most discriminating set of features (DNAm-DEGs) from CD in an independent set of patients from a publicly available therapy response cohort of 20 CD patients with peripheral blood gene expression data (external validation cohort, see the “Methods” section) [14]. Our model was able to predict therapy outcome in the external validation cohort with an accuracy of 85% (Table 3) and a formal prediction model built using the DNAm-DEGs in this cohort obtained an area under the ROC curve of 0.88 (Fig. 6G). In addition, we observed significant correlations in the regulation of DNAm-DEGs across time points and therapy responses between the two cohorts (Spearman’s rho = 0.52) (Fig. 6H).

**Table 3:** Statistics of prediction model testing using the validation cohort

Statistic	Value	Standard error (bootstrap with 100 replicates)
Accuracy	0.85	0.07
Accuracy 95% CI	0.62–0.97	
Sensitivity	1.00	0
Specificity	0.50	0.2
Positive prediction value (PPV)	0.82	0.08
Negative prediction value (NPV)	1.00	0

## Discussion

Here, we performed a longitudinal multi-omics study on blood samples collected from 14 IBD patients receiving infliximab therapy (discovery cohort) at 7 time points (from baseline to 14 weeks after therapy induction) to identify dynamic molecular signatures associated with clinical remission or non-remission to anti-TNF therapy at week 14. It is clear from IBD and other inflammatory diseases that high-resolution omics technologies in blood can unravel important insights into disease trajectories and identify meaningful biomarkers [14, 18, 46–49]. Although it may not fully reflect the local mechanism of action of anti-TNF treatment, we have chosen peripheral blood as a primary analyte as it represents the most routinely available biospecimen in clinical practice. The drastic alterations in transcription at the early stages of the treatment were specific to infliximab treatment while later changes were more likely to be shared between TNF antagonist and anti-integrin treatment, possibly reflecting general mucosal healing processes. Transcriptomic (RNA sequencing) and DNA methylation (genome-wide bead array) signatures obtained from the discovery cohort were confirmed in a replication cohort consisting of 23 independent IBD patients undergoing treatment with TNF antagonists. Selected features of the molecular signatures were able to predict therapy outcome in another independent cohort using an independent method (expression array).

Several previous studies have suggested individual markers, e.g., oncostatin M serum levels [15, 50] and sTREM-1/TREM1 mRNA levels [9, 51] as *ex ante* predictors of anti-TNF response. The classical approach of analyzing signatures of IBD patients before therapy did not identify a valid set of *ex ante* markers in the prospective two-cohort approach. It is important to state that the two cohorts were recruited at the same center using the same clinical criteria and endpoints and identical profiling technologies. Inflammatory characteristics (e.g., clinical activity or CRP) and global molecular signatures between discovery and validation cohorts were not significantly different. Also, after combining the two cohorts and stratifying for disease entities (CD vs. UC), we could not identify robust disease-specific baseline signatures for therapy response. Given the limited cohort size, our finding of course does not rule out the potential presence of such biomarkers. In particular, we cannot exclude the influence of potential covariates, e.g., age or gender, which might become overt in a larger patient population. Conversely, many of the early molecular changes associated with primary response or non-response were shared among the two cohorts. Antagonizing TNF led to dampening of numerous inferred processes (e.g., T cell polarity, neutrophil chemotaxis/extravasation) in circulating immune cells even in patients who did not achieve remission at week 14. Separate gene expression analysis of CD and UC patients showed a T cell-specific signature, which was unique to CD remission patients. A TH2/eosinophil signature, which is inhibited by anti-IL5R (benralizumab) treatment in the peripheral blood of asthma patients [52] is upregulated in both CD and UC patients who do not attain remission at week 14. This signature is consistent with the hypothesis of an underlying type II immunity in non-responders which could be aggravated by blocking TNF. The result corroborates earlier findings on an aggressive disease behavior and lower anti-TNF persistence in patients with high peripheral blood eosinophil levels [53, 54]. Higher-order gene regulation analysis using transcriptional network construction [30] was able to identify modules of co-expressed transcripts which were disrupted through effective therapy in remitting patients from both cohorts. For network construction, we only used differentially expressed genes as we specifically aimed to identify transcriptional networks, which are regulated upon treatment induction. We therefore note that this approach is not completely unbiased. One of the modules (M7) identified in this analysis comprised a cluster of type I interferon-induced genes, which is consistent with earlier hypothesis-based findings in rheumatoid arthritis and IBD, where high type I IFN signatures correlated with poor response to TNF antagonists. A second module (M12) was enriched for transcripts involved in hematopoiesis and platelet aggregation, which is in line with clinical observations that anemia and a pro-coagulative state are important components of the inflammatory response in IBD [55–57]. Indeed, effective anti-TNF therapy has been linked to these processes in other chronic inflammatory diseases outside of the gastrointestinal tract, e.g., rheumatoid arthritis, psoriatic arthritis, and ankylosing spondylitis [58–61], yet this association is less clear in IBD [62]. Thus, in addition to following up on sophisticated molecular marker sets, it might thus be interesting to formally analyze early changes in erythropoiesis and platelet activation markers and their association with clinical response to anti-TNF treatment.

Differential DNA methylation was used as a layer of information to understand potential longer-term regulatory events and cell type distribution linked to anti-TNF non-response. Again, we could not find consistent baseline differences between the discovery and the replication cohort, whereas early longitudinal signatures separating patients with clinical remission at week 14 and non-responders were significantly conserved. Using physical neighborhood [21] for intersecting DNAm changes with DEGs, we show a clear association of an inflammatory neutrophil signature with favorable clinical outcome, corroborating earlier findings that subacute inflammation as a negative predictor of therapy outcome in IBD [63].

We used different sets of omics markers (DEGs, modules, DMPs, DMP-modules, and DMP-linked DEGs) for machine-learning-based feature selection to assess the predictive potential of early changes (baseline vs. week 2) in each data layer. In this step, performance could be increased by using individual disease entities (CD vs. UC) and DMPs and combined DMP-DEGs were the best performing initial data sets for UC and CD, respectively. Features selected from other data moieties, e.g., from co-expression modules, had a lower predictive power compared to DNAm-DEGs at this early time point of therapy. Using the DMP-DEG-based transcriptome features to classify an independent cohort of infliximab-treated CD patients with publicly available expression data, we were clearly able to discriminate between responders and non-responders, although slightly different response criteria (e.g., CDAI instead of HBI) were applied between the studies, indicating the robustness of our marker set. Several previous studies have tested clinical parameters such as CRP and disease activity scores at baseline as predictors of response to anti-TNF agents in CD and UC patients [64]. In our study, prediction models built on clinical parameters at baseline and week 2 were clearly inferior to the ones built on molecular datasets, suggesting that molecular profiling may substantially improve therapy response prediction.

Limitations of our study include the small size of the cohorts due to the dense longitudinal sampling scheme of IBD patients, which we aimed to compensate by rigorous replication of findings in the two-cohort setting. Potential confounding effects of different sequence batches were minimized by randomization of patients across sequencing runs while keeping all longitudinal samples from a given individual in a single batch. As TNF inhibition is similarly effective in CD and UC and is likely a distant intervention into the pathophysiology, we hypothesized that potential mechanisms of action and non-response are at least partially conserved among the disease sub-entities. We therefore deliberately combined CD and UC patients in many analyses to increase power. Although we found congruent molecular signatures of anti-TNF treatment in our combined analysis of IBD patients, entity-specific analyses and feature selection results raise the notion that for further translation into clinical studies, distinct sets of markers might be needed for each IBD subentity. Our prediction models were built using the combined data set using a cross-validation approach and only the CD model could be further validated in an external, publicly available data set. Thus, we cannot rule out overfitting of the model and our initial findings mandate further prospective validation. In this study, we have used validated activity scores (partial Mayo score, HBI) as clinical remission endpoints. Future studies are warranted combining molecular assessments with more objective parameters such as endoscopy and histology, which could further improve outcome prediction aiming at optimal disease control.

## Conclusions

In summary, our study focused on the dynamics of molecular changes occurring shortly after the induction of a targeted anti-cytokine therapy and their association to clinical outcome at week 14. The (ex-post) molecular signature identified includes features and biological processes such as type I interferon signaling, erythropoiesis, and platelet aggregation that are elicited by the impact of the targeted intervention and could be involved in inducing disease control as the ultimate success of such treatment in IBD. We propose that early shifts of immunological network states of circulating blood cells after a first probatory administration of the drug, i.e., ex-post signatures, could carry important information that might guide clinical decision-making such as intensifying or early switch of treatment. Our results in IBD could serve as a blueprint for immune-mediated inflammatory disorders in general to create personalized therapeutic strategies with the aim of making tailored therapeutic choices to achieve disease control.

## Supplementary Information

**Additional file 1: Table S1.** Sampling scheme and samples used for the study.

**Additional file 2: Figure S1.** Baseline signatures of the discovery cohort. **Figure S2.** Transcriptomic changes in response to therapy and induction of remission in the discovery cohort. **Figure S3.** DNA methylation patterns in response to therapy and induction of remission in the discovery cohort. **Figure S4.** Integration of DNA methylation and transcriptome data. **Figure S5.** Molecular comparisons between discovery and replication cohorts and baseline signatures of replication cohort. **Figure S6.** Replication of molecular signatures. **Figure S7.** Comparison of IBD subtypes.

## Data Availability

The raw and processed RNA-sequencing data generated during this study is available and is accessible at the NCBI GEO website under the accession number GSE191328 (https://www.ncbi.nlm.nih.gov/geo/query/acc.cgi?acc=GSE191328) [23]. The Illumina EPIC Array data generated during this study is also available in GEO under the accession number GSE191297 (https://www.ncbi.nlm.nih.gov/geo/query/acc.cgi?acc=GSE191328) [33]. The custom codes used in this study will be shared on github (https://github.com/Systems-Immunology-IKMB/IFX_therapy_response) [65].

## Abbreviations

TNF: Tumor necrosis factor
IBD: Inflammatory bowel disease
UC: Ulcerative colitis
CD: Crohn’s disease
HBI: Harvey-Bradshaw Index
CRP: C-reactive protein
IL-6: Interleukin-6
WGCNA: Weighted gene co-expression network analysis
DNAm: DNA methylation
DMP: Differentially methylated positions
AUC: Area under the curve
ROC: Receiver operating curve
PCA: Principal component analysis
DEG: Differentially expressed gene
ANOVA: Analysis of variance
kME: Module membership score
IFN: Interferon
