# Supplementary material for: Longitudinal multi-omics analysis identifies early blood-based predictors of anti-TNF therapy response in inflammatory bowel disease
Source: Genome Med. 2022 Sep 24;14:110. doi: 10.1186/s13073-022-01112-z (PMC9509553; doi:10.1186/s13073-022-01112-z)
Supplement: Supplementary file 2 — Additional file 2: Figure S1. Baseline signatures of the discovery cohort. Figure S2. Transcriptomic changes in response to therapy and induction of remission in the discovery cohort. Figure S3. DNA methylation patterns in response to therapy and induction of remission in the discovery cohort. Figure S4. Integration of DNA methylation and transcriptome data. Figure S5. Molecular comparisons between discovery and replication cohorts and baseline signatures of replication cohort. Figure S6. Replication of molecular signatures. Figure S7. Comparison of IBD subtypes. [file 13073_2022_1112_MOESM2_ESM.pdf]

Supplementary Figures for *Longitudinal multi-omics analysis identifies early blood-based predictors of anti-TNF therapy response in inflammatory bowel disease*

Neha Mishra, Konrad Aden, Johanna I. Blase, Nathan Baran, Dora Bordoni, Florian Tran, Claudio Conrad, Diana Avalos, Charlot Jaeckel, Michael Scherer, Signe B. Sørensen, Silja H. Overgaard, Berenice Schulte, Susanna Nikolaus, Guillaume Rey, Gilles Gasparoni, Paul A. Lyons, Joachim L. Schultze, Jörn Walter, Vibeke Andersen, SYSCID Consortium, Emmanouil T. Dermitzakis, Stefan Schreiber, Philip Rosenstiel

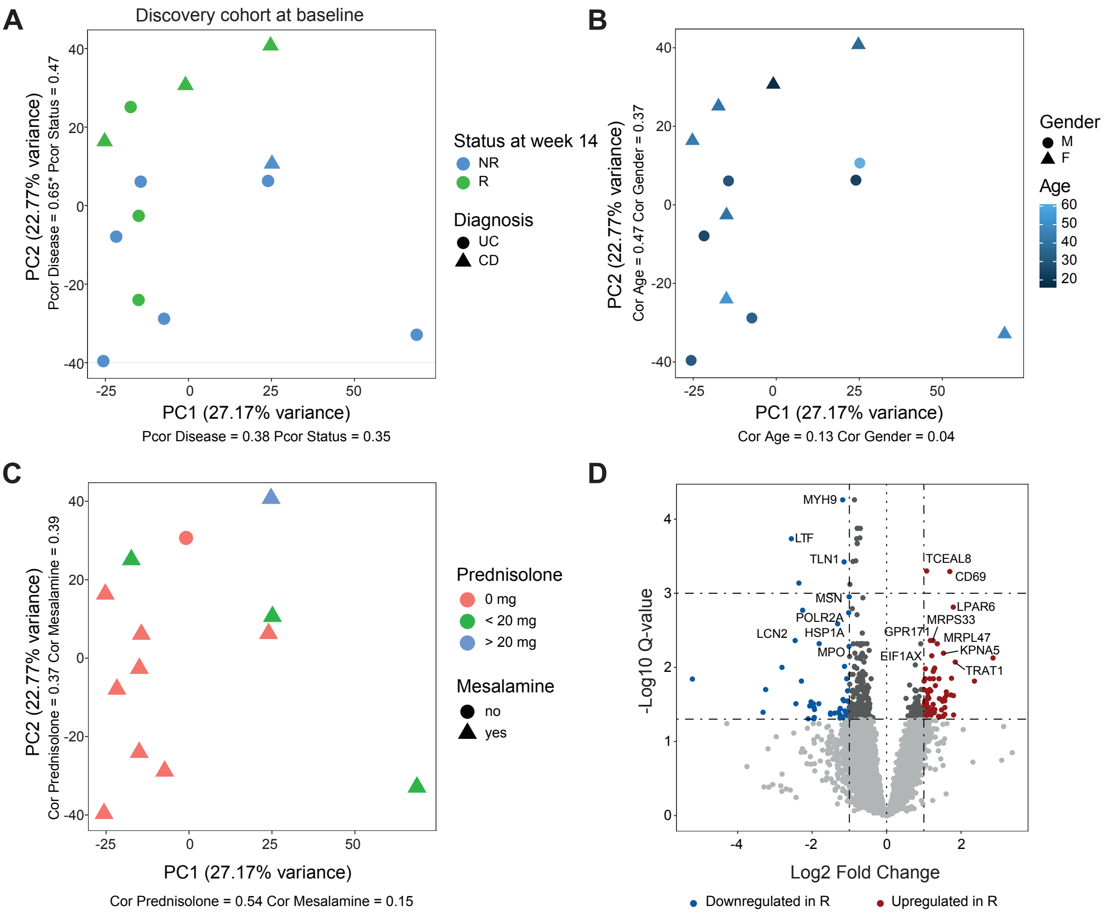

Fig. S1. Baseline signatures of the discovery cohort. A. PCA plot of baseline samples from the discovery cohort based on the expression of all genes. Samples are colour-coded by the remission status at week 14 and shapes correspond to different diagnoses. **B.** PCA plot of baseline samples from the discovery cohort based on the expression of all genes. Samples are colour-coded by the age of the patients and shapes correspond to their gender. **C.** PCA plot of baseline samples from the discovery cohort based on the expression of all genes. Samples are colour-coded by the Predisolone intake of the patients and shapes correspond to the status of the Mesalamine therapy. **D.** Volcano plot depicting log fold changes and FDR adjusted *p* values comparing gene expression between remitters and non-remitters at baseline. Up- and downregulated genes in remitters are highlighted in red and blue, respectively and selected genes are marked.

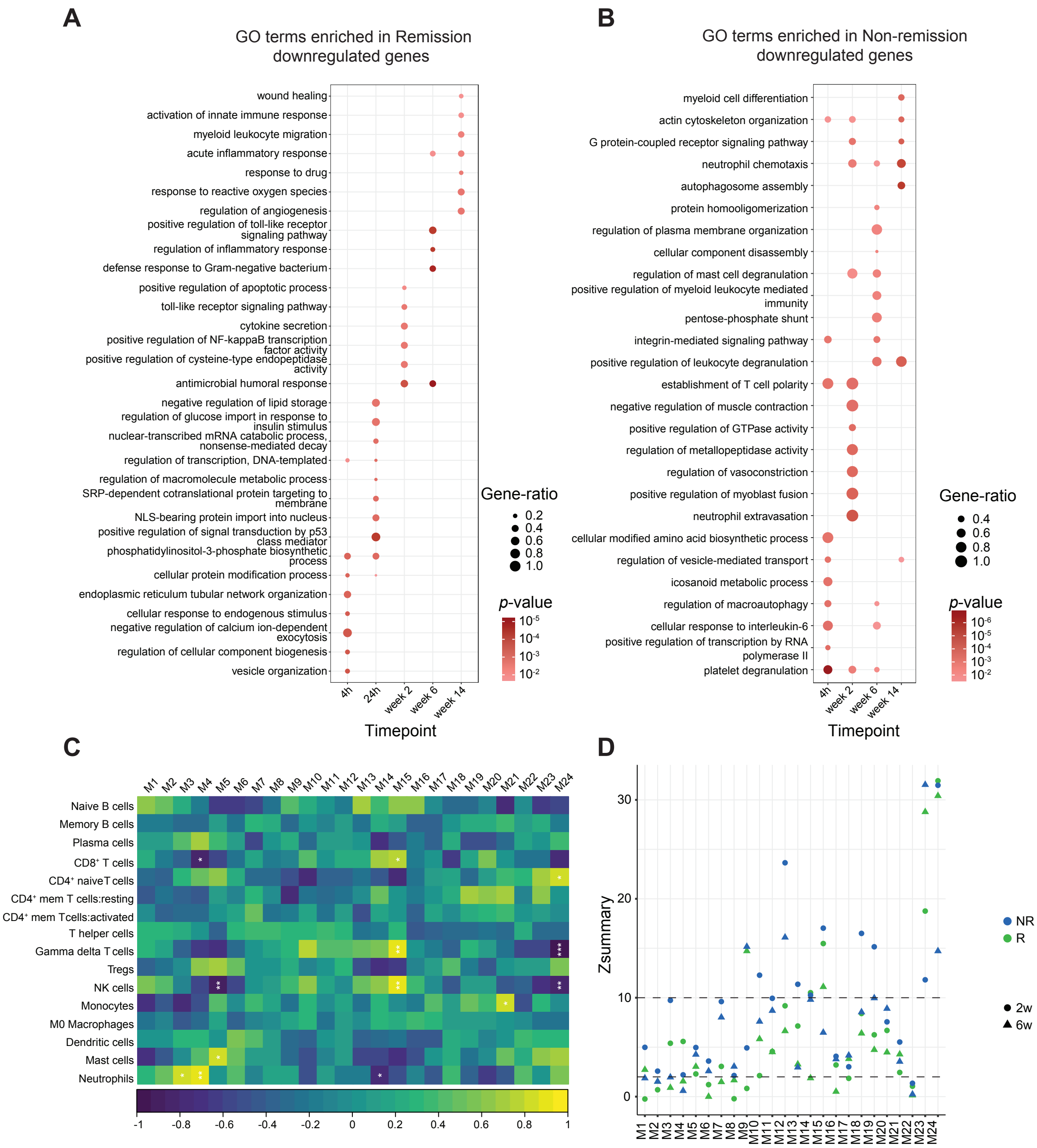

Fig. S2. Transcriptomic changes in response to therapy and induction of remission in the discovery cohort. A-B. GO terms enriched in downregulated genes across timepoints compared to baseline in patients who attained remission at week 14 (**A**) and patients who did not attain remission (**B**). The size of the dots represents the fraction of genes within the GO category that is enriched, and the color of the node represents the p-value of the enrichment. **C.** Correlation heatmap showing Spearman's rank correlation between gene co-expression modules (rows) and inferred cell-type proportions from RNA-seq data (columns). *p < 0.05, ** p < 0.01, and *** p < 0.001 in Spearman's correlation. **D.** Graph showing the Zsummary scores of baseline co-expression modules in remission (green) and non-remission (blue) samples at 2 (circle) and 6 (triangle) weeks in the discovery cohort.

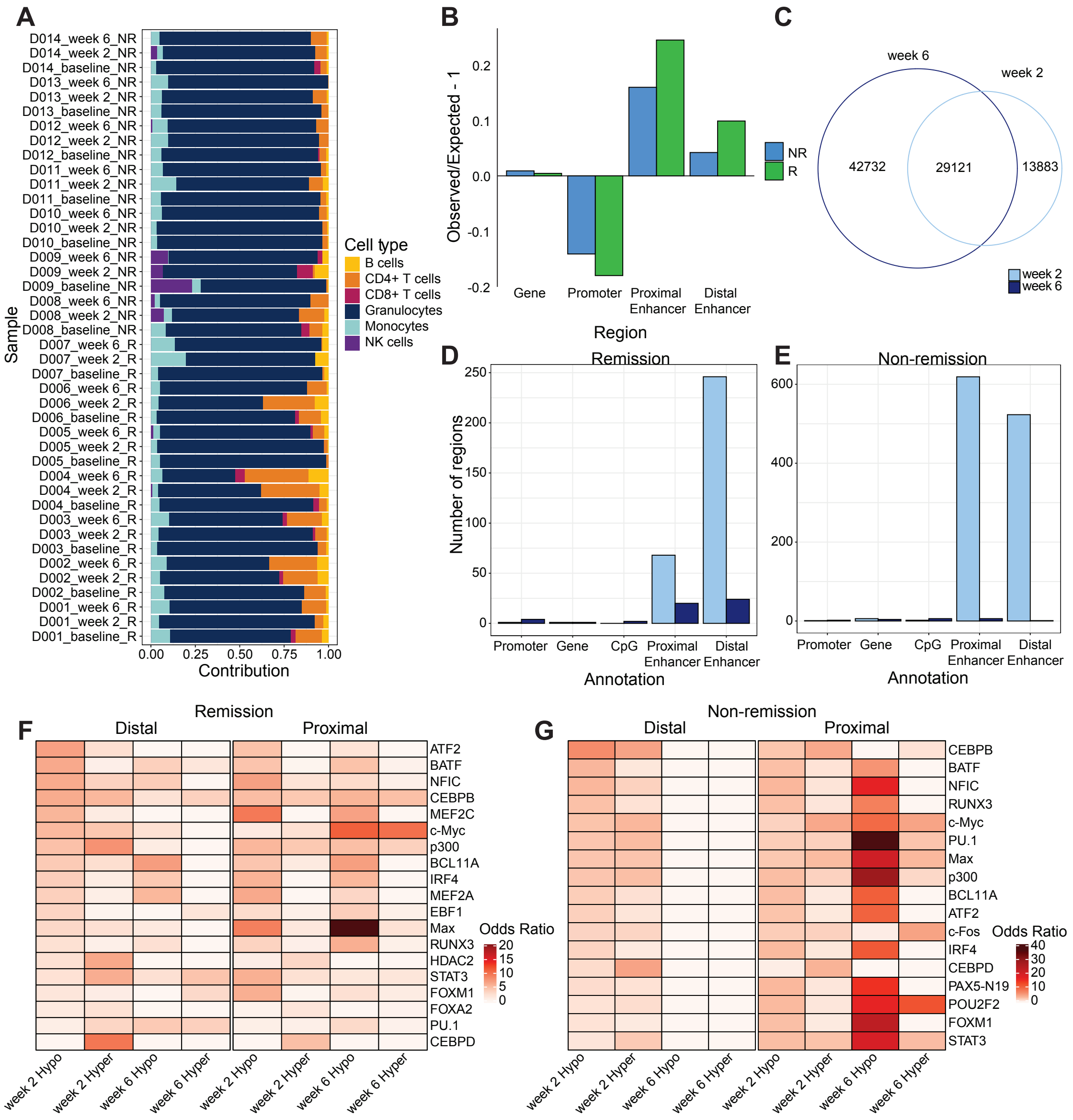

Fig. S3. DNA methylation patterns in response to therapy and induction of remission in the discovery cohort. A. RnBeads computational deconvolution of whole blood samples from discovery cohort based on EPIC array data. **B.** Over-representation and under-representation of differentially methylated positions (DMPs) in genomic regions. The over-/under-representation is quantified as the ratio of observed and expected number of DMPs present in each genomic region under the Chi-square distribution. **C.** Venn diagram showing the number of DMPs at 2 (light blue) and 6 (dark blue) weeks in patients that achieve remission at week 14. **D-E.** Number of differentially methylated regions (DMRs) in remission (**D**) and non-remission (**E**) patients at 2 (light blue) and 6 (dark blue) weeks after therapy induction obtained from the pairwise analysis of the discovery cohort. **F-G.** Heatmaps showing the significant enrichment, quantified by odds ratio, of transcription factor binding sites (TFBS) in differentially methylated distal and proximal enhancer regions identified at different at 2 and 6 weeks after therapy in remission (**F**) and non-remission (**G**) patients. Selected top TFs are visualized.

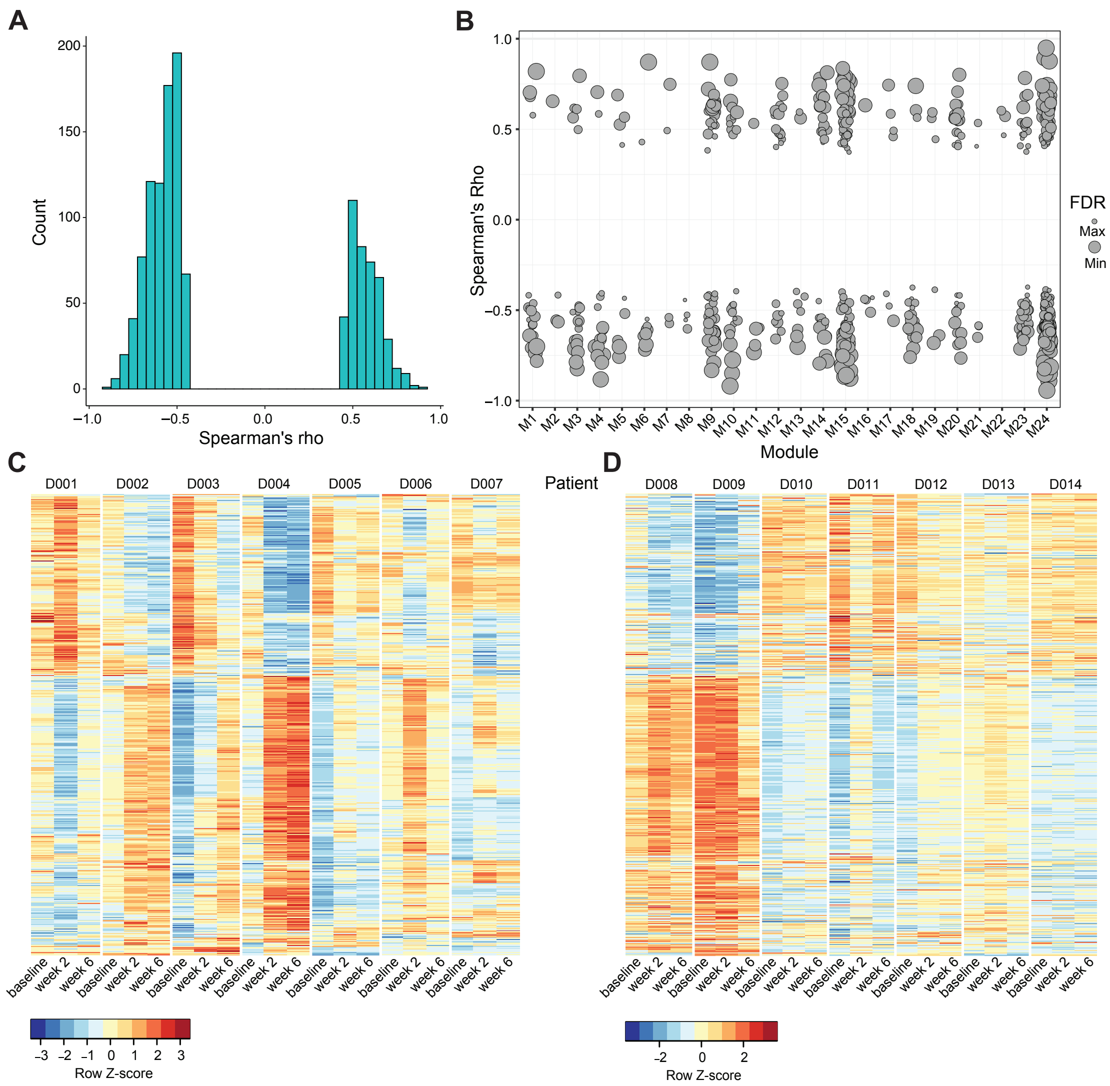

Fig. S4. Integration of DNA methylation and transcriptome data. A. Distribution of Spearman's correlation coefficient between DEGs and their associated DMPs in patients who attained remission at week 14 in the discovery cohort. DMP-DEG correlations with FDR < 0.05 are visualized. **B.** Correlation between co-expression module genes and their nearby DMPs. Each point represents a gene, and the size of the points is proportional to statistical significance (FDR) of the correlation with larger points being more significant. DMP-DEG correlations with FDR < 0.05 are visualized. The positions of individual points are jittered horizontally to show the density of the data. **C-D.** Heatmaps of DMPs, which are correlated with DEGs, showing the scaled methylation intensities at each time point in remission (**C**) and non-remission (**D**) patients.

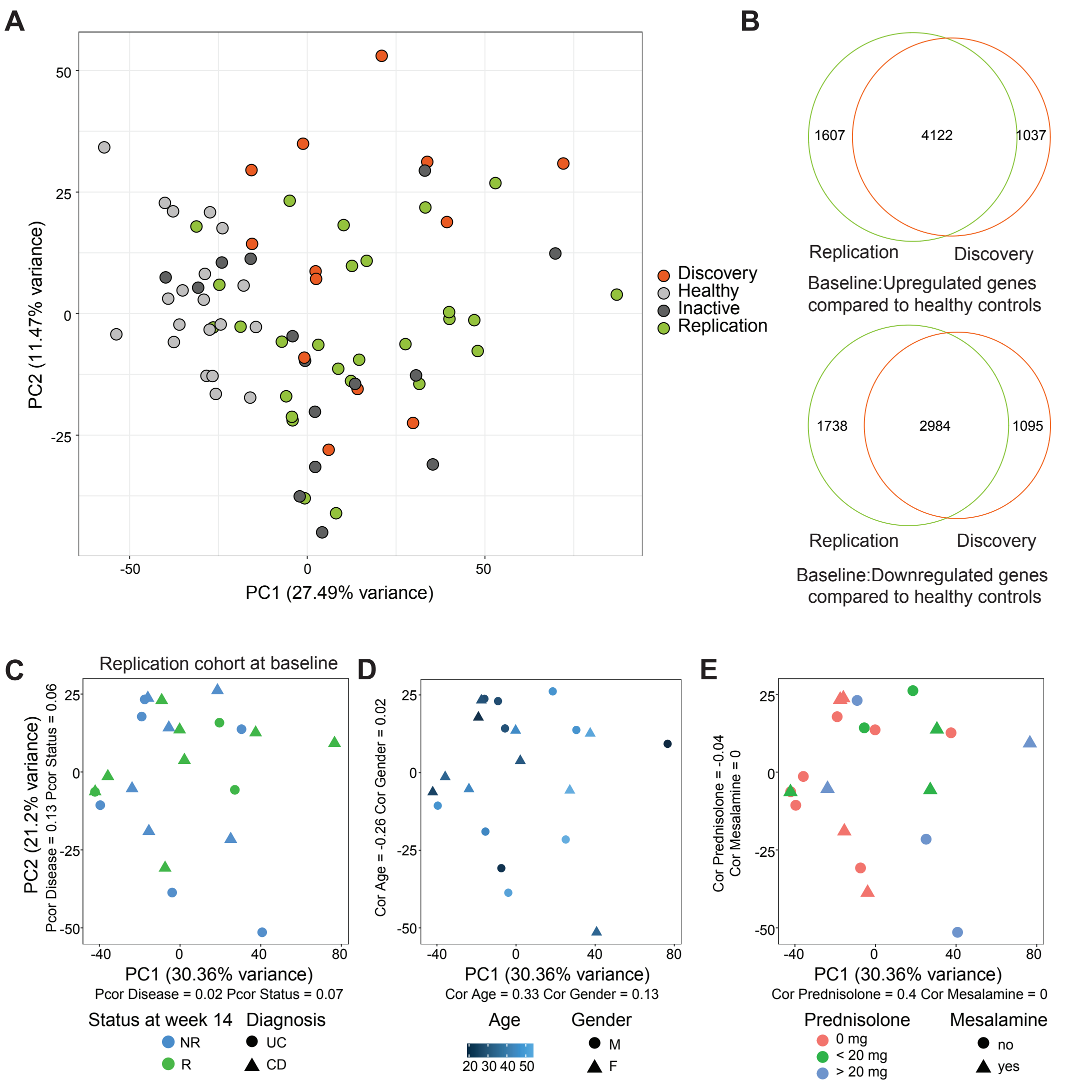

Fig. S5. Molecular comparisons between discovery and replication cohorts and baseline signatures of replication cohort. A. PCA plot of baseline samples from the discovery and replication cohort along with samples from healthy controls and patients with inactive disease based on the expression of all genes. Samples are colour-coded by the cohort and disease activity status. **B.** Venn diagrams showing upregulated (top) and downregulated (bottom) genes in the baseline samples from the discovery (orange) and replication (green) cohort compared to healthy controls. **C.** PCA plot of baseline samples from the replication cohort based on the expression of all genes. Samples are colour-coded by the remission status at week 14 and shapes correspond to different diagnoses. **D.** PCA plot of baseline samples from the replication cohort based on the expression of all genes. Samples are colour-coded by the age of the patients and shapes correspond to their gender. **E.** PCA plot of baseline samples from the replication cohort based on the expression of all genes. Samples are colour-coded by the Predisolone intake of the patients and shapes correspond to the status of the Mesalamine therapy

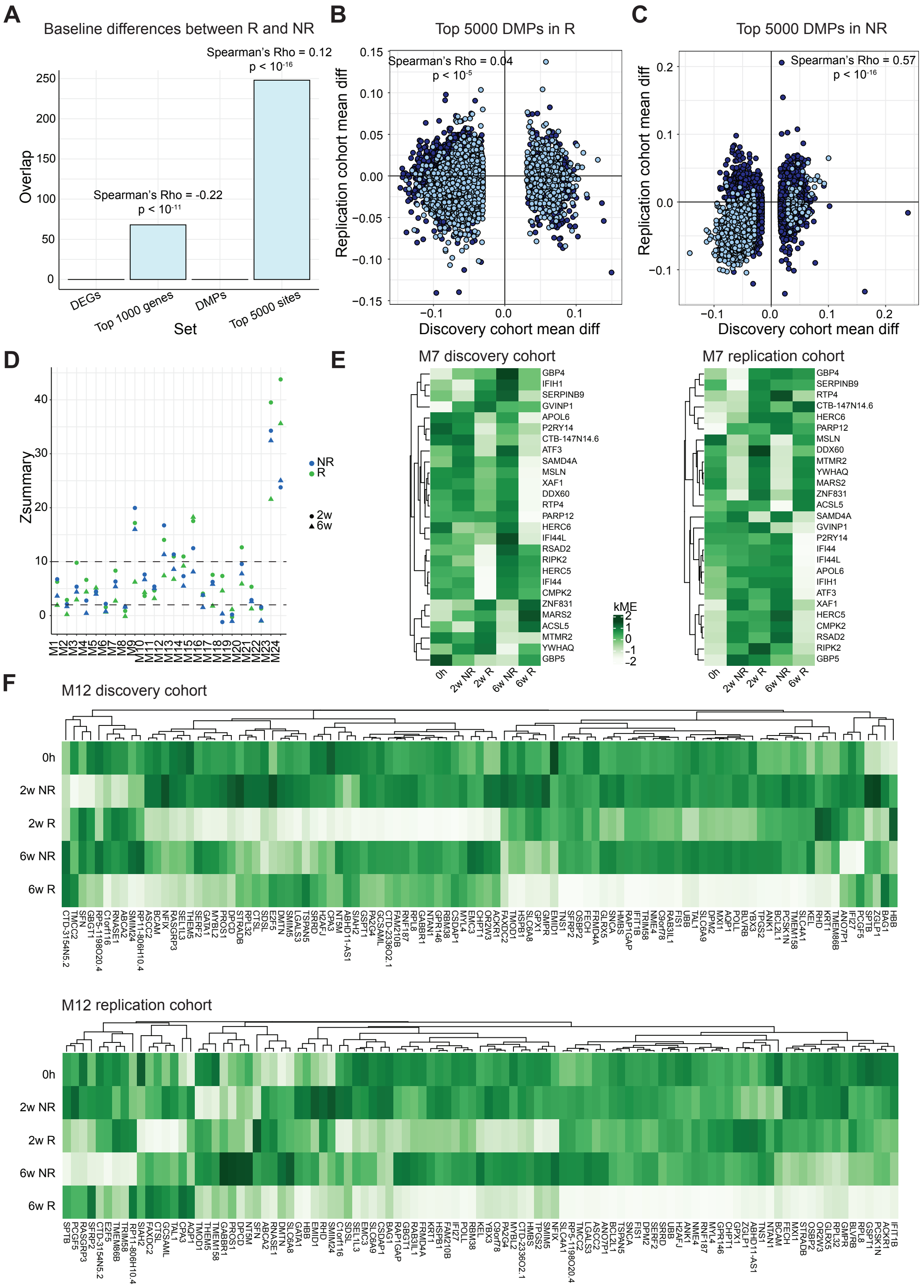

Fig. S6. Replication of molecular signatures. A. Bar plot showing numbers of DEGs and DMPs between remitters and non-remitters at baseline in the discovery cohort that are also differentially expressed/methylated in the replication cohort. The correlations between the log fold change of genes/mean methylation difference of sites are depicted in the plot. **B-C.** Comparison between the mean methylation difference of top 5000 DMPs in remission (**B**) and non-remission (**C**) patients at week 2 (light blue) and 6 (dark blue) in discovery and replication cohorts. **D.** Graph

showing the Zsummary scores of baseline co-expression modules from the discovery cohort in remission (green) and non-remission (blue) samples at 2 (circle) and 6 (triangle) weeks in the replication cohort. **E-F.** Heatmaps showing the module membership score (kME) of M7 (**E**) and M12 (**F**) at different timepoints in discovery and replication cohorts.

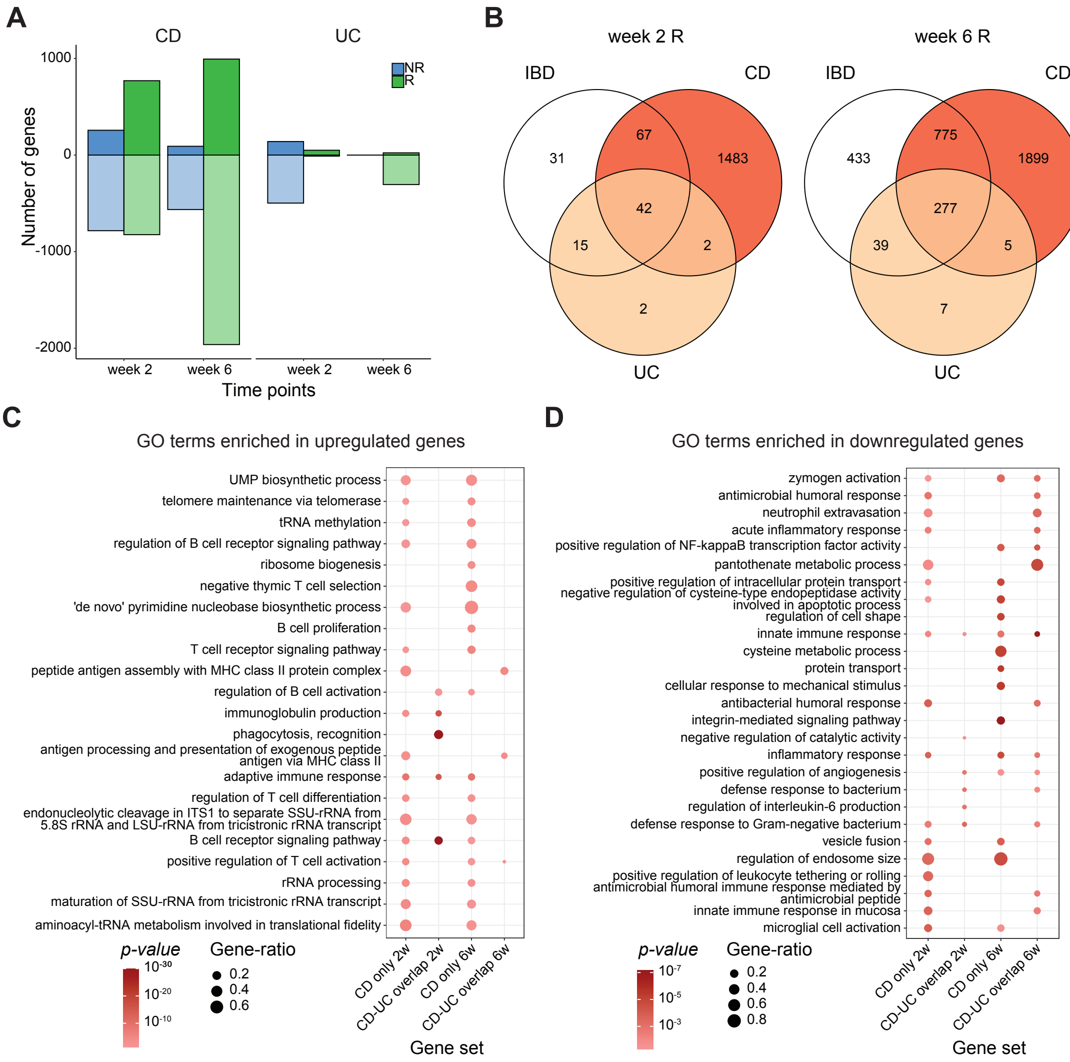

Fig. S7. Comparison of IBD subtypes. A. Number of up-regulated (dark) and down-regulated (light) genes in remission (green) and non-remission (blue) patients at 2 weeks and 6 weeks after therapy induction obtained from the pairwise analysis of CD and UC patients of the combined (discovery and replication) cohort. Negative numbers are used to show number of downregulated genes. **B.** Venn diagrams showing the overlap between differentially expressed genes at week 2 (left) and week 6 (right) obtained from the analysis of IBD patients of the discovery cohort and CD and UC patients of the combined cohort who attained remission at week 14. **C-D.** GO terms enriched in upregulated (**C**) and downregulated (**D**) genes that are either unique to CD or overlap between CD and UC. The size of the dots represents the fraction of genes within the GO category that is enriched, and the color of the node represents the p-value of the enrichment.
